# Prediction of Mechanical Properties of Highly Functional Lightweight Fiber-Reinforced Concrete Based on Deep Neural Network and Ensemble Regression Trees Methods

**DOI:** 10.3390/ma15196740

**Published:** 2022-09-28

**Authors:** Sergey A. Stel’makh, Evgenii M. Shcherban’, Alexey N. Beskopylny, Levon R. Mailyan, Besarion Meskhi, Irina Razveeva, Alexey Kozhakin, Nikita Beskopylny

**Affiliations:** 1 Department of Unique Buildings and Constructions Engineering, Don State Technical University, Gagarin Sq. 1, 344003 Rostov-on-Don, Russia; 2 Department of Engineering Geology, Bases, and Foundations, Don State Technical University, 344003 Rostov-on-Don, Russia; 3 Department of Transport Systems, Faculty of Roads and Transport Systems, Don State Technical University, 344003 Rostov-on-Don, Russia; 4 Department of Roads, Don State Technical University, 344003 Rostov-on-Don, Russia; 5 Department of Life Safety and Environmental Protection, Faculty of Life Safety and Environmental Engineering, Don State Technical University, 344003 Rostov-on-Don, Russia; 6 Department of Mathematics and Informatics, Faculty of IT-Systems and Technology, Don State Technical University, Gagarin sqr., 1, 344003 Rostov-on-Don, Russia; 7 Department Hardware and Software Engineering, Faculty of IT-Systems and Technology, Don State Technical University, 344003 Rostov-on-Don, Russia

**Keywords:** artificial intelligence methods, artificial neural network, deep learning, ensemble method, regression, lightweight fiber-reinforced concrete

## Abstract

Currently, one of the topical areas of application of artificial intelligence methods in industrial production is neural networks, which allow for predicting the performance properties of products and structures that depend on the characteristics of the initial components and process parameters. The purpose of the study was to develop and train a neural network and an ensemble model to predict the mechanical properties of lightweight fiber-reinforced concrete using the accumulated empirical database and data from construction industry enterprises, and to improve production processes in the construction industry. The study applied deep learning and an ensemble of regression trees. The empirical base is the result of testing a series of experimental compositions of fiber-reinforced concrete. The predicted properties are cubic compressive strength, prismatic compressive strength, flexural tensile strength, and axial tensile strength. The quantitative picture of the accuracy of the applied methods for strength characteristics varies for the deep neural network method from 0.15 to 0.73 (MAE), from 0.17 to 0.89 (RMSE), and from 0.98% to 6.62% (MAPE), and for the ensemble of regression trees, from 0.11 to 0.62 (MAE), from 0.15 to 0.80 (RMSE), and from 1.30% to 3.4% (MAPE). Both methods have shown high efficiency in relation to such a hard-to-predict material as concrete, which is so heterogeneous in structure and depends on many factors. The value of the developed models lies in the possibility of obtaining additional useful information in the process of preparing highly functional lightweight fiber-reinforced concrete without additional experiments.

## 1. Introduction

Currently, there is a need to introduce methods of digitalization of business and technological processes, including in the construction industry, which helps construction companies move towards the introduction of modern information technologies. There is a tendency to accumulate and digitize the available data with the aim of further applications of mining algorithms [1,2,3,4,5] to predict the properties of materials, which will further optimize the construction process at all stages, including quality control in the production of building materials.

The modern construction industry, and in particular the production of complex composite materials such as concrete, is facing a serious problem that often affects the quality of buildings and structures being built, leads to an increased number of accidents in construction and, in general, significantly reduces the life cycle of buildings and structures [6,7,8,9]. All of this requires innovative approaches, not only at all stages of the life cycle of buildings and structures but also at the stage of concrete production itself, that is, even at the stage of construction industry enterprises [6,7,8,9]. The problems, in turn, arising in the enterprises of the construction industry are expressed in manufacturing defects and the imperfection of prescription and technological factors that affect the quality of the resulting concrete, as well as in factors such as the influence of the human factor, errors in the selection of compositions, the production of concrete, and their assessment in terms of the ratio of initial parameters and output parameters. In this regard, it also implies the digitalization of all branches of modern industry, the application of artificial intelligence in the construction industry, and in particular, in concrete technology, which contains many vectors and directions for digitalization and improvement of properties, is seen as a relevant direction [10,11,12,13,14,15,16]. Currently, one of the relevant areas among artificial intelligence methods in industrial production is neural networks, which allow one to create systems for predicting output parameters, that is, the operational properties of any products, structures, buildings and structures that depend on the characteristics of the initial components and process parameters. All this shows that the production of concrete can be improved using artificial intelligence methods, as well as the development, training, and use of special neural networks to determine the characteristics of the resulting concrete [17,18,19,20,21]. A brief overview of such methods is presented in Table 1.

In the study [19], predictive models were developed to analyze and predict the crack width in the junction zone of a reinforced concrete beam and a column subjected to lateral cyclic loads. Four machine learning models were developed to predict the width of cracks in seven nodes of reinforced concrete beams and columns. The results showed that the support vector machine–dot kernel (SVM-dot kernel) model can provide accurate performance of the fracture width prediction process. The discrepancy between the measured and predicted fracture width were 30%, which turned out to be smaller compared to DL-max-out (56%), DL-rectifier (55%), and SVM-neural (48%). However, this model is only applicable for lateral cyclic loading simulating seismic loading and needs to be improved in the case of different types of reinforced concrete [19].

In a comparison of multilayer perceptron neural networks (MLPNNs), adaptive neural systems for fuzzy detection (ANFIS), and genetic programming (GEP) in terms of predicting compressive and tensile strengths, the highest efficiency was noted for GEP and the lowest for MLPNN. The order of accuracy for the compressive and tensile strength models is GEP > ANFIS > MLPNN. The advantage of GEP is that it provides a new mathematical equation that can be used to predict the properties of another database. Sensitivity analysis showed that water and cement are the determining factors in the development of the compressive strength model. However, these factors have the least impact on the development of a tensile strength model [20].

Backpropagation neural network (BPNN) models have been used to predict the torsional strength of reinforced concrete beams [22], the unconfined compressive strength of high-strength concrete [23] showed high accuracy, and the resulting BAS-BPNN model with the beetle antennae search (BAS) algorithm outperformed widely used machine learning models such as SVM, random forest (RF), K-nearest neighbors (KNN), logistic regression (LR), and multiple-linear regression (MLR) [23,24].

The Convolutional Neural Network (CNN) has found applications in the construction industry, especially for monitoring and inspection purposes, in particular, to identify areas covered by fresh and young concrete [25] and predict the corresponding concrete damage class [20]. A novel approach to automatically extract defects in high-precision concrete surface images by combining the advantages of image processing and the deep convolutional neural network (DCNN) and attempting to evaluate concrete quality based on detection results has also shown good performance [26].

A method for predicting the 28-day compressive strength of concrete using Multilayer Feed-forward Neural Networks (MFNNs), based on the inadequacy of existing methods dealing with multiple variables and non-linear problems, demonstrates that using a neural network to predict the strength of concrete is practical and beneficial [27,28].

A comparison of several methods, such as artificial neural networks, the support vector machine, classification and regression trees, the Gaussian regression process, and the extreme gradient boosting tree in terms of predicting the properties of fiber-reinforced concrete, showed the effectiveness of the latter, which had the least error. The tree model with an extreme gradient was the best imitator in predicting the properties of fiber-reinforced concrete [29].

A study of the structural behavior of hollow concrete columns (HCC) reinforced with fiberglass, which performed detailed numerical simulations, is presented in [30]. The authors proposed a design-oriented stress–strain model that can capture the softening and curing behavior of FRP-reinforced HCCs [30].

Depending on the actual production situation and performance requirements, it is proposed to choose one of the methods for developing high-strength concrete (UHPC) with the given requirements. Integrating two or more methods can serve as a good approach to addressing the challenges of UHPC development by taking advantage of the benefits of each method [31].

All the above-mentioned machine learning methods and neural network models have been applied to a wide range of materials, such as heavy concrete without additives [9,27,28], concrete with the addition of industrial and agricultural waste ash [21], slag [16], eggshell powder [12], microsilica [20], recycled concrete aggregate [18,32], ceramic waste [11], reinforced concrete with the addition of carbon nanotubes/nanofibers, [10] high-strength concrete [23,31], fiber-reinforced concrete [29], reinforced concrete (columns, beams, slabs) [17,19,22,30], and geopolymer concrete [13], as well as porous cement pastes [33], soil with cement [14], and metals [15].

However, it is known that the properties of lightweight concrete are much more difficult to predict than the properties of normal mass concrete, especially when the prediction concerns the thermal insulation properties of concrete with artificial lightweight aggregate (LWA) [34,35]. The presented results prove that the additional benefits of using ANN include the ability to design lightweight concrete composition with high accuracy, optimize the composition, and predict the properties of the cement composite both in terms of compressive strength and determining the coefficient of thermal conductivity of the modulus of elasticity. Furthermore, the correct use of ANN for the design of new structures made of lightweight concrete should be preceded by laboratory tests, since the results of the studies are extremely important for a complete understanding of the design process of such structures [34,35,36,37,38,39,40,41].

As a result of experiments in the production of highly functional lightweight fiber-reinforced concrete, researchers have generated a large stream of data containing important information about the mechanical properties of the resulting material. Data such as the volume content of components, experimental results, description of experimental results (the class of concrete and its strength and density), and others often have an unstructured and complex form (in the form of texts in natural language, tables, and graphs). The use of methods for the intelligent processing of accumulated data arrays will allow for structuring data, automating the solution of many problems that arise in practice, thus improving the quality of construction production technology, and optimizing costs by reducing the time required to determine the key properties of the building material in question.

The scientific novelty lies in:

- Revealing the fundamental possibility of predicting the properties of lightweight fiber-reinforced concrete using artificial intelligence methods.
- Determination of the quantitative and qualitative picture of the relationship between the initial data of the formulation and technology and the output parameters expressed in the properties of concrete.
- The fundamental basis of structure formation and the formation of concrete properties, presented for the analysis of the constructed neural network and the issuance of applied forecasts to it on the operational characteristics of concrete.
- Proof of the possibility of neural network control of the structure and properties of concrete and analysis of complex relationships that were previously inaccessible through the manual method.

In this regard, the main purpose of the study is to develop and train a deep neural network, as well as to use an ensemble regression tree model to determine the mechanical properties of lightweight fiber-reinforced concrete. It is possible to realize the set purpose using the accumulated empirical base and data available at the disposal of the construction industry enterprises. The ultimate purpose of the study is to improve the production processes in the construction industry.

The main task of the study was to achieve the ability to control the properties of concrete using artificial intelligence methods using lightweight fiber-reinforced concrete with improved characteristics as an example. The next task of the authors in the future is to project the result obtained onto other types of concrete and establish the technical feasibility and rational ranges of such control.

The stages of the study are as follows:

- Review and analysis of existing literature in the areas of improving the quality of the concrete industry and the introduction of new management and quality control systems in production, as well as a review of existing authors’ methods for the application of artificial intelligence methods in the concrete industry and manufacturing enterprises in general.
- Selection of an empirical base built on physical experiments and accumulated experience with the results of testing concretes and the mutual dependencies between the input parameters of the initial components and the technological and output parameters of the resulting concretes established by us and other authors.
- After processing and applying the data of a physical experiment, it is necessary to develop, train, and test a deep neural network, as well as apply an ensemble model of regression trees to process the empirical base with a further comparison of the results based on the values of the main metrics.
- Evaluate the prospects of applying the developed methods in practice and evaluate the possibility of translating and projecting the results obtained to other types of concrete, as well as developing specific proposals for the construction industry.

## 2. Materials and Methods

Determination of the key mechanical properties of highly functional lightweight fiber-reinforced concrete, such as the density, cubic compressive strength, prism compressive strength, tensile strength in bending, and axial tensile strength, is of great importance in determining the quality and further performance properties of the material. Ideally, these properties should be obtained empirically. However, it is quite often difficult to carry out appropriate measurements after a series of experiments due to the high cost and time-consuming nature.

The evaluation of the mechanical properties of highly functional lightweight fiber-reinforced concrete is an area in which artificial intelligence methods can be very effective due to saving material resources and time. In this work, the following methods will be applied: Deep learning and an ensemble of regression trees. The methods discussed represent an alternative way to obtain reliable and accurate results to determine the mechanical properties of a material, since there can be both linear and non-linear relationships between input data (features) and output (predicted) values.

### 2.1. Materials

The initial dataset for the methods under consideration is the results of 153 experimental compositions for concrete with strength ranging from 40 to 50 MPa (Table S1). The features of the models are the content of glass fiber in an amount from 0 to 8% (with a step of 0.5) from the mass of cement and the way the fibers are distributed (1t—pre-mixing of cement, water + mixing with fiber + mixing with sand and crushed stone; 2t—pre-mixing of cement, sand, crushed stone + mixing with fiber + mixing with water; 3t—pre-mixing of cement, sand, crushed stone, water + mixing with fiber). At the same time, the properties and their values are constant for all variables. The predicted parameters are cubic compressive strength (MPa), prismatic compressive strength (MPa), flexural tensile strength (MPa), and axial tensile strength (MPa). Data collection on testing lightweight fiber-reinforced concrete was carried out by the authors of the study using their own accumulated empirical database and data from construction industry enterprises. Lightweight fiber concrete samples were tested in accordance with GOST 10180 “Concretes. Methods for Strength Determination Using Reference Specimens” [42] and GOST 24452 “Concretes. Methods of prismatic compressive strength, modulus of elasticity and Poisson’s ratio determination” [43] according to the research program presented in [36].

Table 2 presents the main statistical characteristics of the analyzed dataset.

Here, the “glass fiber content” and “fiber distribution method” data are feature values (training sets), and the cubic compressive strength, prismatic compressive strength, flexural tensile strength, and axial tensile strength are prediction value data (prediction sets).

### 2.2. Deep Learning

**Figure 1:**
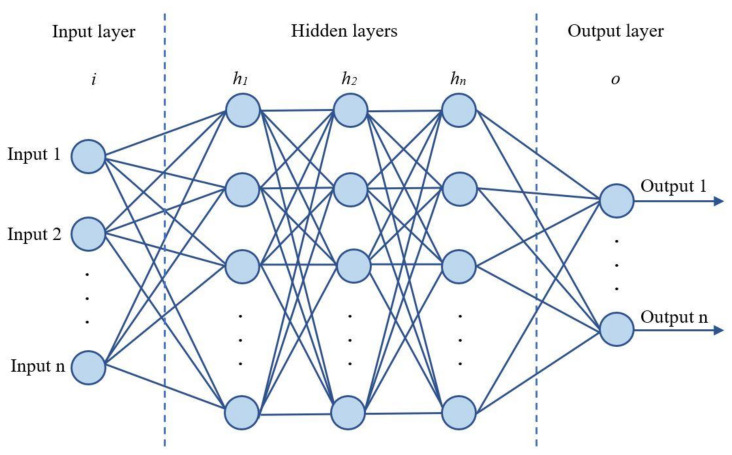
Deep neural network architecture.

The concept of “deep learning” describes the training of so-called “deep artificial neural networks”. The architecture of a deep artificial neural network is a structure (Figure 1) consisting of a large number of internal (hidden) layers with adjustable parameters—the weight coefficients of artificial neurons that make up each layer of the network. In the first input layer (input layer, *i*), the network receives a vector of features that describe the object—in this case, two features. In the inner layers (hidden layer, *h*_1_ … *h*_n_), they are processed: The input vector is multiplied by the matrix of connections, and the vector of new features formed in this way is transferred to the next layer. The signal processing result is sent to the output layer of the network (Output layer, o).

For the study, a neural network of the following architecture was selected (Table 3): A fully connected neural network of direct propagation, consisting of 5 hidden layers containing 40, 30, 20, 30, and 40 neurons, respectively. As the activation function on the hidden layers, the most commonly used activation function in deep learning is Relu. As an optimization algorithm, the quasi-Newtonian algorithm of Broyden–Fletcher–Goldfarb–Shannot with limited use of Limited-memory BFGS (LBFGS) memory was used. To prevent overfitting, the early stop form of regularization was used.

The choice of neural network architecture (number of neurons, layers, nature of connections) was justified by a small empirical base. In such cases, a network with an excess number of elements would lose its generalization ability and would work well only on the training set.

### 2.3. Ensemble of Regression Trees

An ensemble of regression trees is a predictive model consisting of a combination of decision trees, each of which by itself gives a low quality of regression, but due to their large number, the result is satisfactory, thereby combining regression trees increases predictive efficiency.

The study used an ensemble model of regression trees with a variety of parameters to select the best ones (Table 4).

The Number of Trees parameter specifies the total number of decision trees to create in the ensemble. Their number can vary from a few dozen to several thousand. Typically, an ensemble with a good predictive score requires several hundred to several thousand “weak learners”. However, in the case of small samples, one should start with a few dozen trees, evaluate the efficiency of the ensemble, and then, if necessary, increase their number. Another important hyperparameter that needs to be tuned is the depth of decision trees. The study will evaluate the effectiveness of depth from 1 to 3 levels.

## 3. Results

To solve the problem, the MATLAB 9.11 (Release 2021b) environment was used, which implements a deep neural network and the ensemble method of regression trees.

The following metrics were used to assess the quality of the constructed models: Mean Absolute Error (MAE), Mean Square Error (MSE), Root-Mean-Square Error (RMSE), and Mean Absolute Percentage Error (MAPE).

(1) $$MAE=\frac{1}{n}\sum_{i=1}^{n}\left|y_{i}-{\hat{y}}_{i}\right|$$

(2) $$MSE=\frac{1}{n}\sum_{i=1}^{n}{\left(y_{i}-{\hat{y}}_{i}\right)}^{2}$$

(3) $$RMSE=\frac{1}{n}\sqrt{\sum_{i=1}^{n}{\left(y_{i}-{\hat{y}}_{i}\right)}^{2}}$$

(4) $$MAPE=\frac{1}{n}\sum_{i=1}^{n}\left|\frac{y_{i}-{\hat{y}}_{i}}{{\hat{y}}_{i}}\right|\times 100$$

where $y_{i}$ is the actual measured value of the quantity in question; ${\hat{y}}_{i}$ represents its predicted value.

When training the neural network, the dataset was divided into training, validation, and test sets in the ratio of 70/10/20. For an ensemble of regression trees, the sample is divided into training and test in the ratio of 80/20; in turn, 10% of the test data, when building trees, go to validation.

### 3.1. Results for a Deep Neural Network

Consider the process of training a deep artificial neural network when predicting the parameter “cubic compressive strength” (MPa).

**Figure 2:**
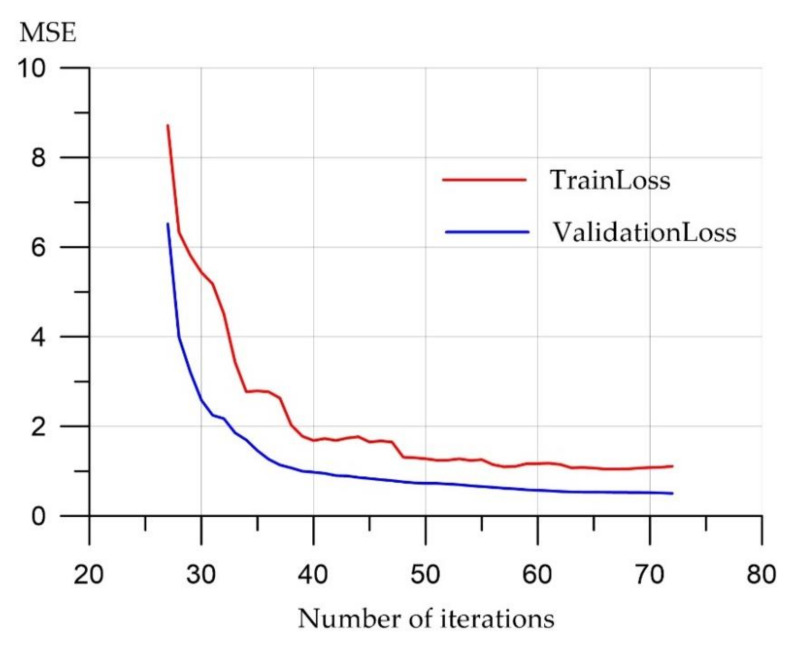
Neural network training.

From the graph (Figure 2), which shows the process of training the neural network, it can be seen that the error in the training and validation sets tends to be 0, which indicates that this architecture is suitable for approximating experimental data.

### 3.2. Results for an Ensemble of Regression Trees

It makes sense to build graphs of errors against the number of trees, taking into account the depth of the tree structure, and limit the size at the moment when the errors become the smallest.

**Figure 3:**
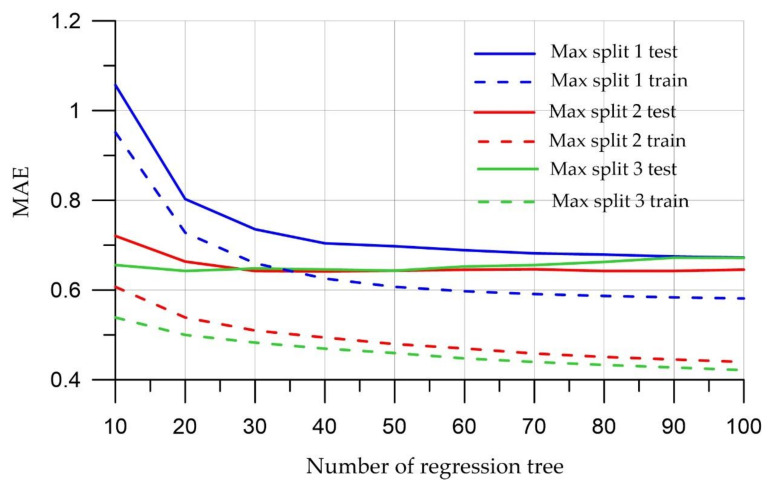
Mean Absolute Error meaning.

**Figure 4:**
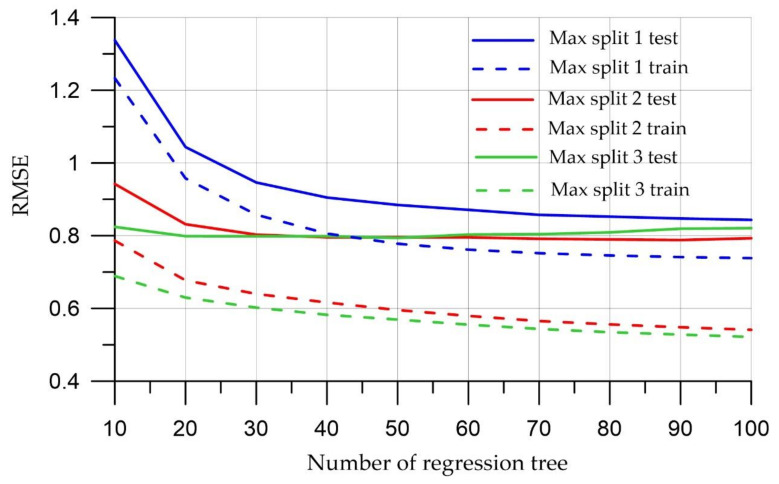
Meaning of Root-Mean-Square Error.

**Figure 5:**
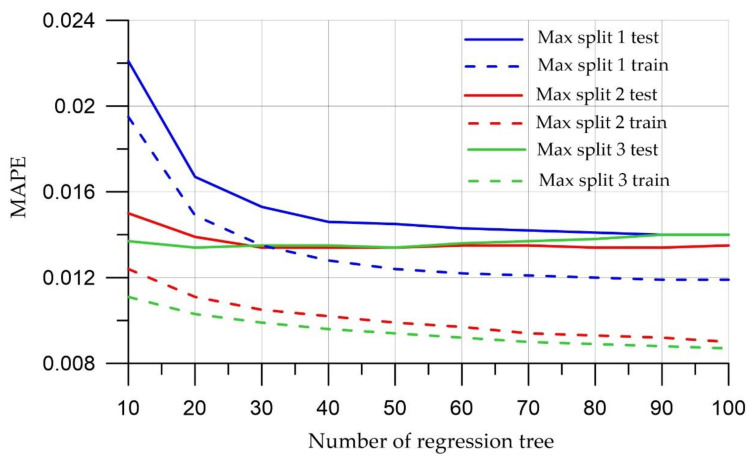
Mean Absolute Percentage Error.

Figure 3, Figure 4 and Figure 5 show the dynamics of the above metrics for different tree depths with the number of regression trees in the ensemble ranging from 10 to 100 for test and training sets.

Figure 3, Figure 4, Figure 5 show that a large number of trees does not guarantee the high quality of the model; however, it was noticed that the model running time increases when this parameter increases. The most optimal value for the number of trees is 10, since at this value, both the training and test samples have the smallest error values. In terms of tree depth, trees that are too deep can cause models to be too detailed and not generalize to new data. On the other hand, trees that are too small can lead to overly simple models that define data specifics. The depth of the tree is critical, and as Figure 3, Figure 4 and Figure 5 increase, there is an improvement in training and test quality metrics. This study uses a maximum depth of 3 to capture the specifics of a particular dataset.

That is why, according to the figures, the smallest errors are observed when the model runs with a tree depth of 3 and the number of trees is equal to 20.

**Figure 6:**
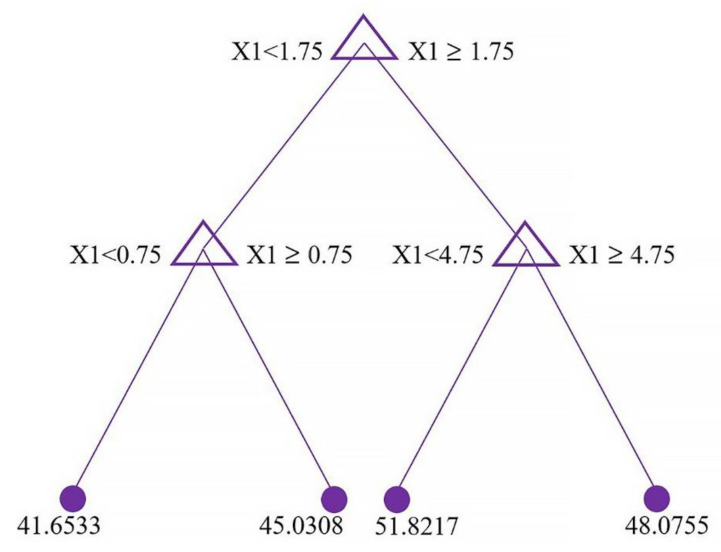
Visualization of the first trained regression tree.

Decision trees allow one to obtain easily interpretable models, which are a set of rules of the form “if … then …”. Interpretation is facilitated, among other things, by the ability to present these rules in the form of a visual tree structure. Figure 6 is a plot of the first trained regression tree for the best model.

**Figure 7:**
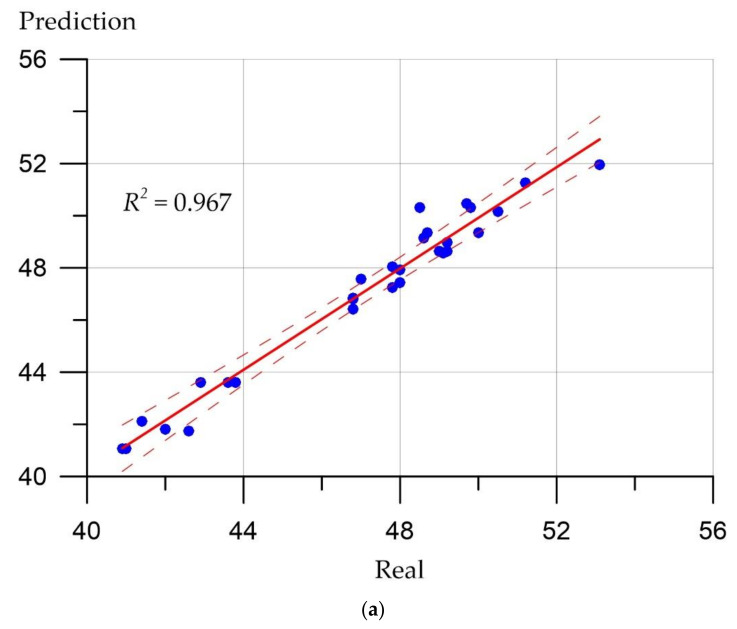

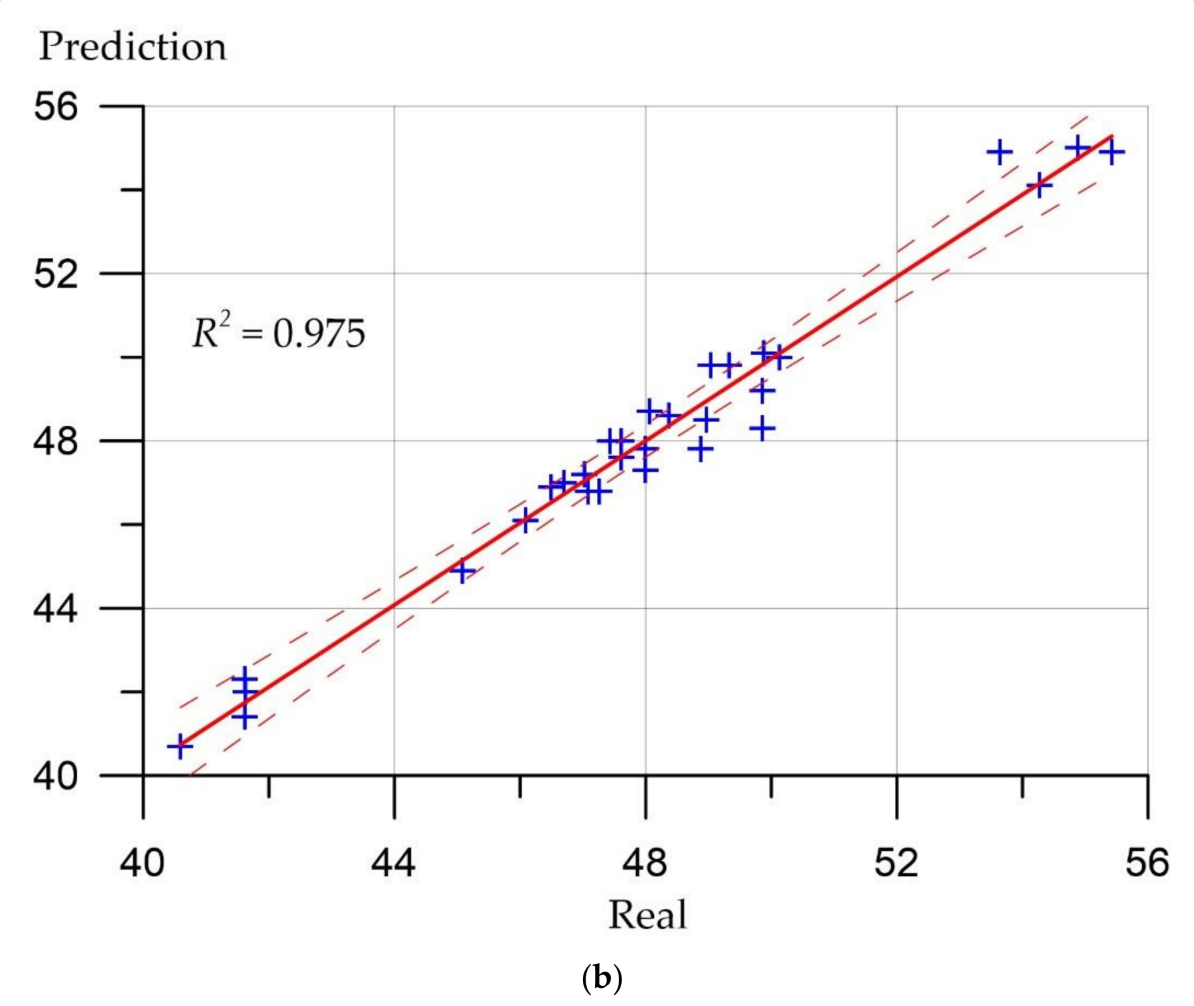
Correlation between real values of cubic compressive strength (MPa) and calculated values: (**a**) Deep neural network; (**b**) ensemble of regression trees.

**Figure 8:**
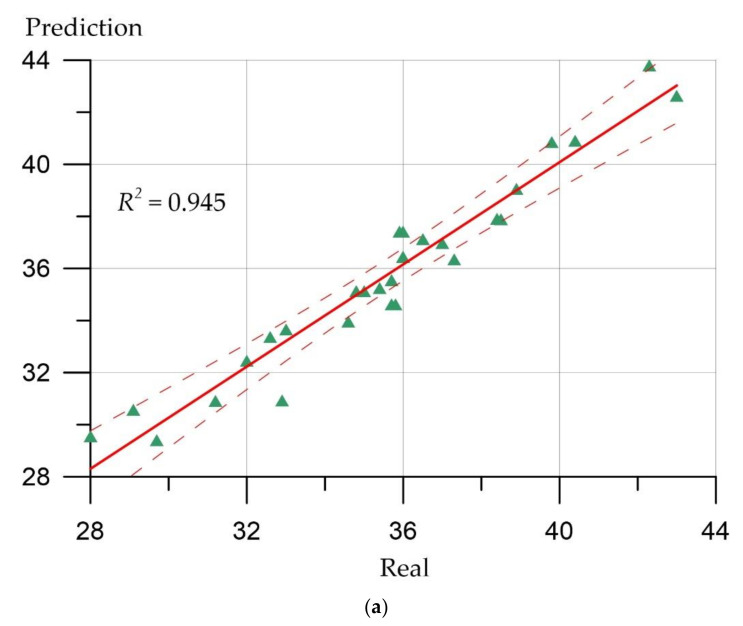

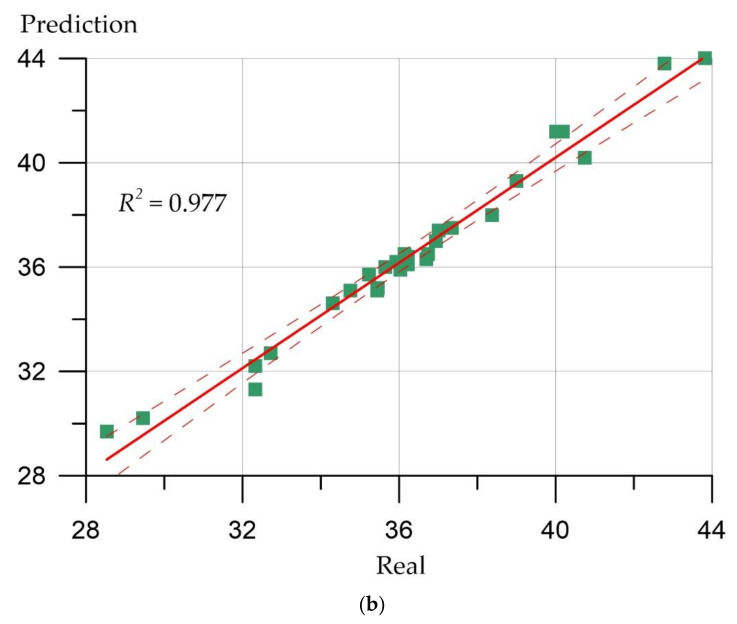
Correlation between real values of prismatic compressive strength (MPa) and calculated values: (**a**) Deep neural network; (**b**) ensemble of regression trees.

**Figure 9:**
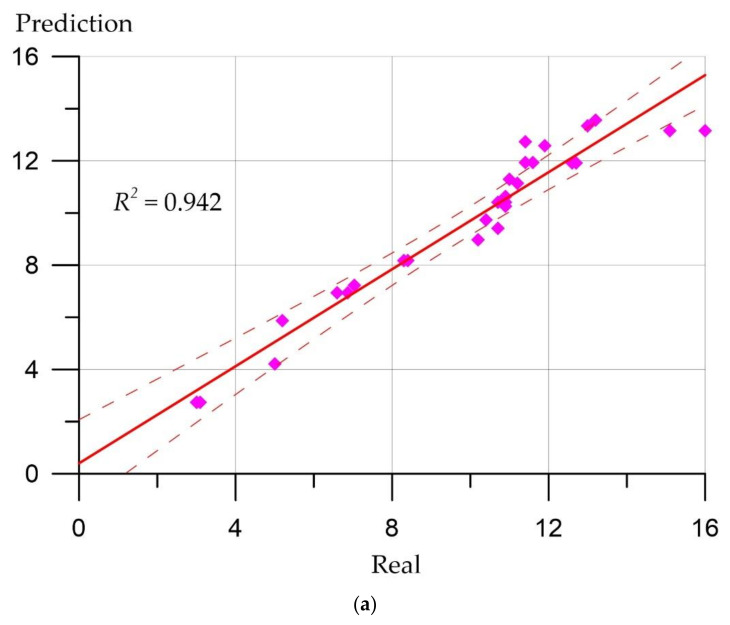

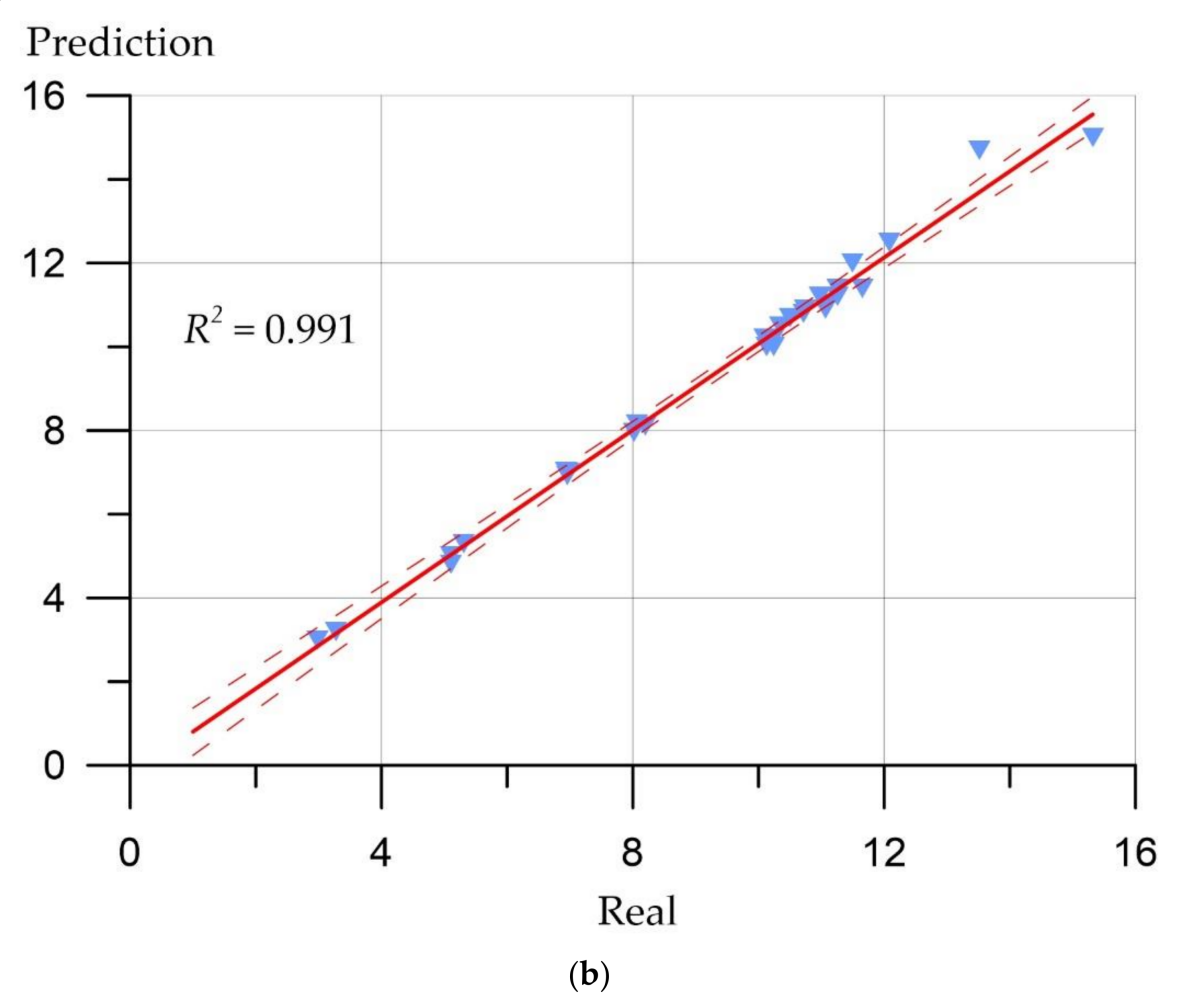
Correlation between real values of tensile strength in bending (MPa) and calculated values: (**a**) Deep neural network; (**b**) ensemble of regression trees.

**Figure 10:**
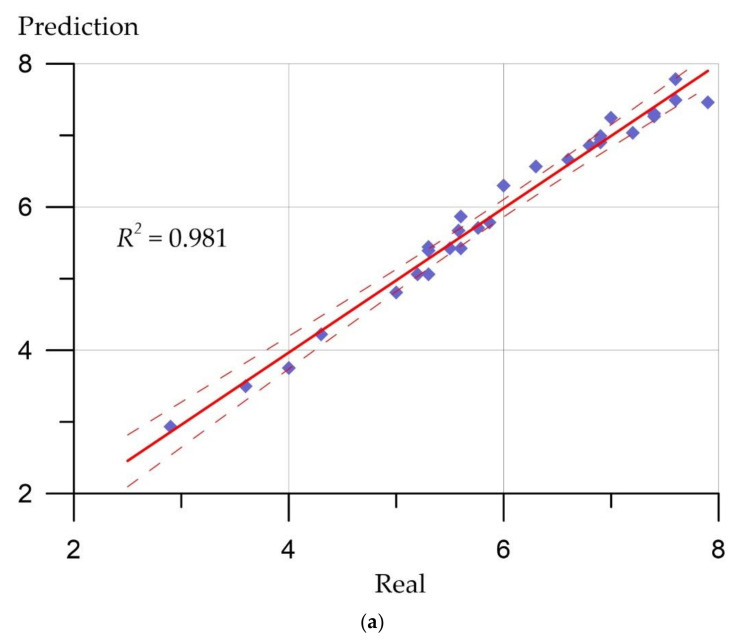

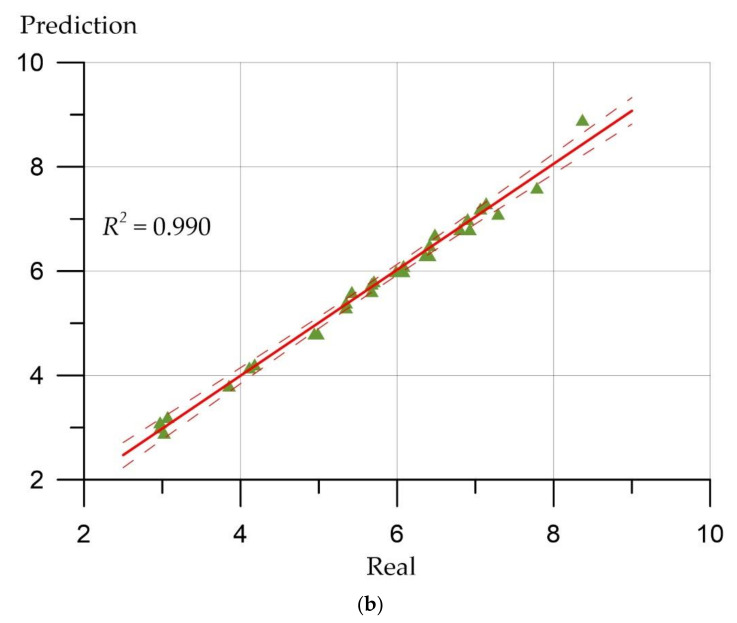
Relationship between real values of axial tensile strength (MPa) and calculated values: (**a**) Deep neural network; (**b**) ensemble of regression trees.

Prediction error plots (Figure 7, Figure 8, Figure 9 and Figure 10) show the actual values from the analyzed data set compared to the predicted values generated by our models. This allows us to see how large the variance is in the model.

The developed regression models can be evaluated using similar graphs, where a line at an angle of 45 degrees is highlighted, and the ratios between the real values of the predicted parameters and their calculated values are presented. Figure 7, Figure 8, Figure 9 and Figure 10 allow us to make sure that the developed models are adequate since the distribution of points on the graphs is free from any patterns and looks like a random spread of points around zero. The coefficient of determination takes values from 0.94 to 0.99, which indicates a high fit of the model to the data.

Table 5, Table 6, Table 7 and Table 8 present a comparative performance of the two methods. Comparing the two considered models, it can be noted that, in some cases, the ensemble method is ahead of the neural network trained using the deep learning method.

The ensemble method of regression trees in this implementation demonstrated the best values of metrics compared to the neural network, since it is a more complex algorithm in terms of the number of parameters than the neural network. Thus, the ensemble method of regression trees had more opportunities to adjust to the data.

## 4. Discussion

As the experience of the application of machine learning methods shows, the use of algorithms for combining models—ensembles—is one of the most powerful methods of machine learning and often surpasses other models in quality. In this study, the ensemble method, which consists of using a committee of decision trees, demonstrated better metric values compared to a neural network due to the ability to work with categorical variables (the fiber distribution method parameter), as well as fine-tuning the key parameters of the model.

Artificial intelligence methods make it possible to generalize the results of experiments for complex models with many parameters (compared to empirical models). It is not necessary to conduct a deep theoretical analysis to obtain an adequate forecast. Even after the derivation of theoretical formulas, their accuracy is often lower than that given by the neural network model. The considered methods are valuable for their ability to generalize the information coming to them during training, and not just memorize specific examples.

Many works are known concerning the application of artificial intelligence methods for various high-tech industries, such as automotive, instrumentation, and various technological industries, as well as the service sector [44,45].

However, in the global space of scientific research and in practice, there is a deficit, expressed in a certain conservatism of the construction industry and the slow pace of the introduction of artificial intelligence methods and other innovative methods in this industry [6,7,8,9,10,11,12,13,14,15,16,17,18,19,20,21,22,23,24,25,26,27,28,29,30,31,32,33,34,35].

The most important parameter of the operational reliability of lightweight concrete is the rational ratio of its strength and density. Due to the fact that the density directly depends on the pore structure and on the whole process of concrete structure formation, the strength indicator comes to the fore. In order to fully use the advantages of fiber reinforcement (or fibers) and rely on the created concrete as a running structural material, a range of strength values from 30 MPa to 40 MPa was adopted in our study. Fiber-reinforced concretes with such strength characteristics have great prospects, and after working out the neural network model on these concretes, it is supposed to project the study to even higher-strength concretes, and also, if necessary, turn to less durable concretes for less critical levels of construction work.

Taking into account the fact that we have previously established objective opportunities to significantly improve the quality of lightweight fiber-reinforced concrete and reduce the percentage of rejects and losses due to the correct recipe and technological approaches, the introduction of artificial intelligence methods in the concrete industry will further improve these processes, achieve a significant increase quality, and ultimately lead to a reduction in the cost of concrete production by up to 20%, which has already been established according to preliminary estimates of industrial partners.

As for scientific deficits, some pioneering studies were carried out earlier in Russia and the CIS, in particular, reference [46], the authors of which used a fuzzy neural network as a model for predicting the strength of reinforced concrete products. The mathematical model showed its effectiveness in testing. The average error was 0.96 MPa or 2% [46].

The quantitative characteristics of the work performed and the result obtained are as follows. We present the characteristics of accuracy for each method for each property. Thus, for the cubic compressive strength, MAE varied from 0.46 to 0.62, RMSE from 0.6 to 0.8, and MAPE from 0.98% to 1.30%. For prismatic compressive strength, the accuracy of the methods was somewhat different: MAE from 0.48 to 0.73, RMSE from 0.62 to 0.89, and MAPE from 1.33% to 2.11%. For bending tensile strength, the accuracy was MAE from 0.30 to 0.63, RMSE from 0.44 to 0.87, and MAPE from 3.40% to 6.62%. Finally, for axial tensile strength, MAE was from 0.11 to 0.15, RMSE from 0.15 to 0.17, and MAPE from 2.06% to 2.49%. Thus, the percentage characteristics of accuracy are very high, and the qualitative picture of the validity and expediency of using the studied methods of artificial intelligence in predicting the properties of concrete is quantitatively confirmed. Such high accuracy is ensured by the accepted large amount of empirical data, in-depth analysis of the results, and, as a result, the good correlation between artificial intelligence methods and standard methods for predicting the strength of concrete. Such high-precision characteristics of the methods are a significant achievement and open further opportunities for the development of the direction of introducing artificial intelligence methods for predicting the properties of concrete.

## 5. Conclusions

The information obtained using the constructed models based on artificial intelligence methods makes it possible to obtain additional information for controlling and adjusting the process of preparing highly functional lightweight fiber-reinforced concrete and obtaining a material with specified strength and density characteristics without additional experiments.

An analysis of the literature data and the results obtained in the course of our own experiments made it possible to formulate the following main conclusions.

(1) Deep neural network and ensemble regression trees methods can be applied to determine the mechanical properties of highly functional lightweight fiber-reinforced concrete.
(2) The developed models are adequate since the distribution of points on the graphs has no patterns and looks like a random scatter of points around zero. The coefficient of determination shows values from 0.94 to 0.99, which indicates a high correspondence of the model to the data.
(3) The ensemble method of regression trees in this implementation demonstrated the best values of metrics compared to the neural network, since it is a more complex algorithm in terms of the number of parameters compared to the neural network. Therefore, the ensemble method of regression trees had more opportunities to adjust to the data.
(4) The quantitative picture of the accuracy of the applied methods for strength characteristics varies: For the deep neural network method, it was from 0.15 to 0.73 (MAE), from 0.17 to 0.89 (RMSE), and from 0.98% to 6.62% (MAPE), and for the ensemble of regression trees, it was from 0.11 to 0.62 (MAE), from 0.15 to 0.80 (RMSE), and from 1.30% to 3.4% (MAPE). Both methods have shown fairly high efficiency in relation to such a hard-to-predict material as concrete, which is very heterogeneous in structure and depends on a large number of factors.
(5) The result obtained in the form of a developed neural network for predicting the properties of lightweight fiber-reinforced concrete allows us to establish the fundamental role of the initial factors, namely the amount of fiber and the method of distribution of fibers in the formation of output parameters—the strength properties of fiber-reinforced concrete. Thus, with the help of the considered methods of artificial intelligence, the role of specific recipe-technological factors, which were previously evaluated only from the point of view of traditional recipes and technology, is revealed. Thus, artificial intelligence methods for building a neural network made it possible to reveal, establish, and manage this fundamental relationship, strengthening the role of the control technologist in the formation and prediction of the properties of specific building materials.

These methods can be easily implemented in the process of development and production of unique building materials, since they do not require serious computing resources, and in the future, based on artificial intelligence, an expert system can be created to summarize all the accumulated experimental data, which can be located in the electronic environment of the university and provide data to interested workers and researchers for the development of the industry. Thus, the main goal of the study was achieved: it was shown that artificial intelligence methods are applicable to this task and have great potential in determining the mechanical characteristics of unique building materials.

In further studies, we plan to focus on the study of new theoretical and practical dependencies that arise in the production of fiber-reinforced concrete made from various components and operated in various conditions, for even greater universalization of the proposed concept.

## Figures and Tables

**Table 1:** An overview of the main artificial intelligence methods used to predict the properties of various types of concrete.

**Reference Number**	**Methods**	**Materials**	**Parameters**		**Objectives**
			**Input**	**Variable**	
[6]	Support vector machine (SVM) Gaussian process regression, ANN	Concrete	P- and S-wave velocities, electrical resistivity, density, water-to-binder ratio	Compressive strength	Recommendation of a set of criteria for evaluating the compressive strength of concrete in a marine environment with various saturation and salinity conditions
[7]	SVM, Adaptive neural fuzzy inference system (ANFIS), ANN	Concrete	Results from the two non–destructive testing tests	Compressive strength	Using non-destructive testing to improve concrete strength estimation by aI predictive models
[8]	Decision tree (DT) Ensemble ML (boosting, Ada Boost)	Fiber reinforced polymer (FRP)	Database of 121 groups of experimental results	Punching shear strength	Build machine learning models to accurately predict the punching shear strength of FRP reinforced concrete slabs
[9]	DT, ANN, Gradient boosting	Concrete	Water, cement, coarse aggregate, fine aggregate, fly ash, microsilica, superplasticizers, nanosilica, temperature	Compressive strength	High temperature compressive strength prediction
[10]	ANN	Self-sensing concrete, carbon nanotubes/carbon nanofibers (CNT/CNF) reinforced concrete	Parameters of the composition of the concrete mixture	Compressive strength, flexural strength	Approximation of the ANN approach to a range of specific researchers and possible implementation of ANN in civil engineering practice
[11]	ANN, DT	Concrete in fresh and hardened states	Dosage of ceramic waste powder 10% and 20%	Compressive strength	Application of ANN and DT to predict compressive strength of concrete containing CWP
[13]	ANN, boosting, Ada Boost ML	Geopolymer concrete (GPC)	Parameters of the composition of the concrete mixture	Compressive strength	Using ANN, Boosting and AdaBoost ML approaches based on Python coding to predict the compressive strength of high calcium fly ash based GPCs
[17]	ANN	Fiber-reinforced polymers—short concrete columns	Column length, modulus of elasticity of fiberglass, compressive strength of concrete, coefficients of longitudinal and transverse reinforcement, ultimate axial load	Load carrying capacity	Forecasting the bearing capacity of fiberglass short concrete columns
[21]	Separate stacking ensemble with the random forest algorithm (SSE-Random Forest) SSE-Bagging, Integrated stacking ensemble (ISE), weighted averaging ensemble (WAE)	Fly Ash Concrete (FAC)	Parameters of the composition of the concrete mixture	Compressive strength	Comparison of ensemble models of deep neural networks, i.e., superlearning algorithm, simple averaging, weighted averaging, integrated summation, as well as individual ensemble summation models and superlearning models. to develop an accurate approach to estimating the FAC compressive strength and to reduce the high variance of the predictive models

**Table 2:** Statistical characteristics of the dataset.

**Variable**	**G** **lass Fiber Content**	**F** **iber Distribution Method**	**Cubic Compressive Strength**	**Prismatic Compressive Strength**	**Flexural Tensile Strength**	**Axial Tensile Strength**
**Unit**	%	-	MPa	MPa	MPa	MPa
**mean**	4.0	-	48.41	36.78	9.41	5.76
**std**	2.46	-	3.98	3.71	3.12	1.56
**min**	0.0	-	40.20	28.0	3.0	2.80
**max**	8.0	-	57.80	44.10	16.0	8.90

**Table 3:** Neural network architecture.

**Parameter Number**	**Parameter**	**Value**	**Description**
1	Network type	Fully Connected Feedforward Neural Network for Solving the Regression Problem	The first fully connected layer of the neural network has a connection from the input of the network, and each subsequent layer has a connection from the previous layer
2	Number of hidden layers	5	1 hidden layer—40 neurons 2 hidden layer—30 neurons 3 hidden layer—20 neurons 4 hidden layer—30 neurons 5 hidden layer—40 neurons
3	Activation function for hidden layers	Relu	$f\left(x\right)=\left\{\begin{array}{c} x,\;\;x\geq 0\\ 0,\;\;x<0 \end{array}\right.$
4	Loss function minimization method	LBFGS	Broyden-Fletcher-Goldfarb-Shannot quasi-Newton algorithm with limited memory usage
5	Regularization method	Early stopping	Scheduled to stop when it starts to deteriorate validation set error

**Table 4:** Parameters of the ensemble model of regression trees.

**Parameter Number**	**Parameter**	**Value**	**Description**
1	Ensemble Aggregation Algorithm	LSBoost	The LSBoost (Least-squares boosting) method uses the least squares method as a loss function
2	Number of trees	10…100 with step 10	The model is a combination of n-decision trees
3	Tree Complexity Level (Depth)	1,2,3	The maximum depth of the tree takes the values 1, 2 and 3
4	Limit on the number of objects in leaves (MinLeafSize)	5	According to the classics, in regression problems it is recommended to use the value 5
5	Minimum number of branch node observations (MinParentSize)	10	Each branch node in the tree has at least the MinParentSize of the observation
6	Learning rate	1	Ensemble model learning rate (can take values in the range (0…1])
7	Method for estimating the generalizing ability of a model	10 block cross validation	10-box cross validation on training data

**Table 5:** Comparative characteristics of the work of the implemented methods on a test sample in determining the cubic compressive strength.

Method	MAE	RMSE	MAPE, %
Deep Neural Network	0.46	0.60	0.98
Ensemble of Regression Trees	0.62	0.80	1.30

**Table 6:** Comparative characteristics of the work of the implemented methods on a test sample when determining the prism compressive strength.

Method	MAE	RMSE	MAPE, %
Deep Neural Network	0.73	0.89	2.11
Ensemble of Regression Trees	0.48	0.62	1.33

**Table 7:** Comparative characteristics of the work of the implemented methods on the test sample when determining the tensile strength in bending.

Method	MAE	RMSE	MAPE, %
Deep Neural Network	0.63	0.87	6.62
Ensemble of Regression Trees	0.30	0.44	3.4

**Table 8:** Comparative characteristics of the work of the implemented methods on the test sample when determining the axial tensile strength.

Method	MAE	RMSE	MAPE, %
Deep Neural Network	0.15	0.17	2.49
Ensemble of Regression Trees	0.11	0.15	2.06

## Data Availability

The study did not report any data.

## Supplementary Materials

The following supporting information can be downloaded at: https://www.mdpi.com/article/10.3390/ma15196740/s1, Table S1: The initial data set.
